# Efficacy, Safety, Tolerability, and Serum IgG Trough Levels of Hyaluronidase-Facilitated Subcutaneous Immunoglobulin 10% in US Pediatric Patients with Primary Immunodeficiency Diseases

**DOI:** 10.1007/s10875-025-01862-6

**Published:** 2025-03-14

**Authors:** Niraj C. Patel, Jolan E. Walter, Richard L. Wasserman, Arye Rubinstein, Suthida Kankirawatana, Meagan W. Shepherd, Erin Greco, Zhaoyang Li, Sharon Russo-Schwarzbaum, Shumyla Saeed-Khawaja, Barbara McCoy, Leman Yel

**Affiliations:** 1 Division of Pediatric Allergy and Immunology, Duke University, Durham, NC USA; 2 University of South Florida, St. Petersburg, FL USA; 3 Division of Pediatric Allergy/Immunology, University of South Florida at Johns Hopkins All Children’s Hospital, St. Petersburg, FL USA; 4 Division of Allergy and Immunology, Massachusetts General Hospital for Children, Boston, MA USA; 5 Allergy Partners of North Texas Research, Dallas, TX USA; 6 Psychiatry Research Institute at Montefiore Einstein, Montefiore Medical Center, Bronx, NY USA; 7 Division of Pediatric Allergy/Immunology, Children’s of Alabama, University of Alabama at Birmingham, Birmingham, AL USA; 8 Marshall University Medical Center, Huntington, WV USA; 9 Takeda Development Center Americas, Inc, Cambridge, MA USA; 10 Baxalta Innovations GmbH, a Takeda company, Vienna, Austria; 11 Division of Basic and Clinical Immunology, Department of Medicine, University of California Irvine, Irvine, CA USA

**Keywords:** Facilitated subcutaneous immunoglobulin, Hyaluronidase, Immunoglobulin replacement therapy, Inborn errors of immunity, Primary immunodeficiencies, rHuPH20

## Abstract

**Purpose:**

To investigate the efficacy, safety, tolerability, and serum IgG trough levels of hyaluronidase-facilitated subcutaneous immunoglobulin (fSCIG) 10% in US pediatric patients with primary immunodeficiency diseases (PIDDs).

**Methods:**

This phase 3, open-label, prospective study (NCT03277313) was conducted at 17 US centers. Eligible patients aged 2 to < 16 years had PIDDs and had received immunoglobulin G (IgG) at a consistent dose for ≥ 3 months before screening. Participants received fSCIG 10% via dose ramp-up for up to 6 weeks (Epoch 1), then every 3–4 weeks for ≤ 3 years (Epoch 2). The primary endpoint was the rate of acute serious bacterial infections (ASBIs).

**Results:**

Data were provided by 44 participants for Epoch 1 (mean ± SD age: 9.0 ± 3.6 years) and 43 (97.7%) for Epoch 2; 34 (77.3%) completed the study. Two ASBIs (both bacterial pneumonia) were reported in one participant with specific antibody deficiency. The mean rate of ASBIs was 0.04 events/participant-year (99% upper confidence interval limit: 0.20), significantly lower than the regulatory-defined threshold of 1.0 (*p* < 0.001). The mean rate of all infections was 3.12 events/participant-year. Stable mean serum IgG trough levels were maintained during Epoch 2 (10.4, 9.2, and 9.2 g/L at Months 0, 6, and 12, respectively). Most related treatment-emergent adverse events were mild or moderate in severity. No participant developed anti-recombinant human hyaluronidase neutralizing antibodies; 1/44 participants (2.3%) developed binding antibodies.

**Conclusion:**

fSCIG 10% effectively prevented ASBIs in pediatric patients with PIDDs, with a favorable safety profile consistent with previous clinical studies.

**Supplementary Information:**

The online version contains supplementary material available at 10.1007/s10875-025-01862-6.

## Introduction

Immunoglobulin replacement therapies administered intravenously (IVIGs) or subcutaneously (SCIGs) are the cornerstone of treatment for most patients with primary immunodeficiency diseases (PIDDs; alternatively referred to as inborn errors of immunity [1]) who do not have the ability to generate and/or maintain adequate antibody responses [2, 3]. Patients with PIDDs are susceptible to chronic and recurrent infections, in addition to having a higher risk of autoimmunity and malignancy than those without PIDDs [4, 5].

SCIG therapies, which offer the potential for patient/caregiver self-administration at home [6], have been adopted over the past 40 years; however, additional therapies continue to be developed [3]. One such therapy, facilitated subcutaneous immunoglobulin (fSCIG) (HyQvia; Baxalta US, Inc., a Takeda company, Lexington, MA, USA), a dual-vial unit of immunoglobulin G (IgG) 10% and recombinant human hyaluronidase (rHuPH20), is approved in the USA for the treatment of PIDDs in adults and, based on the data derived from the study reported herein, is approved in children aged ≥ 2 years [7]. In Europe, fSCIG 10% is approved as immunoglobulin replacement therapy (IgRT) for patients of all ages with PIDDs or secondary immunodeficiency diseases [8]. fSCIG 10% combines the benefits of IVIG and SCIG, with less frequent dosing and fewer systemic adverse reactions than IVIG [9–11], as well as the opportunity of self-administering at home. With a bioavailability greater than 90% of that achieved with IVIG, fSCIG 10% addresses some of the limitations of conventional SCIG, allowing for larger infusion volumes, higher infusion rates, and shorter infusion durations for equivalent doses, with fewer infusion sites required and thus fewer needle sticks for patients [7, 12].

Previous studies, including the pivotal phase 3 study in patients with PIDDs (NCT00814320), demonstrated that fSCIG 10% was effective and could be administered at similar dosing intervals to those used for administration of IVIG, following a dose ramp-up, with fewer systemic adverse events (AEs) reported [12–14]. Further analysis of patients younger than 18 years of age included in the pivotal study and the associated long-term extension also demonstrated low rates of infection, and low rates of local and systemic AEs [13]. In addition, a post-authorization safety study conducted in Europe has provided additional support for the long-term safety of fSCIG 10% in pediatric patients with PIDDs, with a safety and tolerability profile consistent with that observed in previous clinical studies [15].

At the time of this phase 3 study, fSCIG 10% was not yet approved for the treatment of children with PIDDs in the USA (approved April 2023). The objective was thus to further investigate the efficacy, safety, tolerability, immunogenicity and serum IgG trough levels of fSCIG 10% in pediatric patients with PIDDs in the USA.

## Methods

### Study Design

This phase 3, open-label, prospective study (NCT03277313) was conducted at 17 centers in the USA between September 25, 2017 and July 20, 2022.

Patients were eligible for enrollment if they were aged 2 to < 16 years with diagnosis of a PIDD involving an antibody formation defect requiring IgRT, were receiving a consistent dose of IgG for ≥ 3 months before screening, and had a serum IgG trough level of > 5 g/L at screening. Parents/caregivers were required to provide informed consent. Prophylactic systemic antibacterial antibiotics were not permitted during this study. Prophylaxis for viral infections, fungi, parasites (including *Pneumocystis* pneumonia) not treated by immunoglobulins were permitted and recorded as concomitant medications. A full list of study eligibility criteria is included in Table 1.

**Table 1:** Eligibility criteria

Inclusion criteria^a^	Exclusion criteria^b^
• Documented diagnosis of a form of primary immunodeficiency involving a defect in antibody formation and requiring Ig replacement before enrollment (as defined according to the IUIS Scientific Committee 2015 [22]). The diagnosis had to be confirmed by the sponsor’s Medical Director before first treatment with the study drug. • Age ≥ 2 and < 16 years at screening. • Receiving a consistent dose of IgG, administered in compliance with the respective product information for a period of ≥ 3 months before screening. The average minimum pre-study dose was equivalent to 300 mg/kg body weight /4 weeks and the maximum dose was equivalent to 1000 mg/kg body weight/4 weeks. • A serum IgG trough level of > 5 g/L at screening. • If a female of childbearing potential, a negative pregnancy test and agreement to use adequate birth-control measures for the duration of the study. • Participant/legally authorized representative was willing and able to comply with the requirements of the protocol.	• Known history of, or positive at screening for, one or more of the following: hepatitis B surface antigen, PCR for hepatitis C virus, PCR for HIV type 1/2. • Abnormal laboratory values at screening meeting any one of the following criteria (abnormal tests may have been repeated once to determine if they were persistent): o persistent alanine aminotransferase and aspartate aminotransferase > 2.5 times the upper limit of normal for the testing laboratory o persistent severe neutropenia (absolute neutrophil count ≤ 500/mm^3^). • Anemia that precluded phlebotomy for laboratory studies, according to standard practice at the site. • Ongoing history of hypersensitivity or persistent reactions (urticaria, breathing difficulty, severe hypotension, or anaphylaxis) after IVIG, SCIG, and/or immune serum globulin infusions. • Severe IgA deficiency (< 7.0 mg/dL) with known anti-IgA antibodies and a history of hypersensitivity. • Known allergy to hyaluronidase. • Active infection and receiving antibiotic therapy at the time of screening. • A bleeding disorder or a platelet count < 20,000/µL, or, in the opinion of the investigator, at significant risk of increased bleeding or bruising as a result of subcutaneous therapy. • Severe dermatitis that would, in the opinion of the investigator, have precluded adequate sites for safe product administration. • Participation in another clinical study involving an investigational product or investigational device in the 30-day period before enrollment or was scheduled to participate in another clinical study involving an investigational product or investigational device during the course of this study. • Family member or employee of the investigator. • If female, participant was pregnant or lactating at the time of enrollment.

^a^Each participant was required to meet all of the following criteria to be eligible for inclusion in the study

^b^Participants who met any of the following criteria were excluded from the study

*HIV*, human immunodeficiency virus; *Ig*, immunoglobulin; *IgA*, immunoglobulin A; *IgG*, immunoglobulin G; *IUIS*, International Union of Immunological Societies; *IVIG*, intravenous immunoglobulin; *PCR*, polymerase chain reaction; *SCIG*, subcutaneous immunoglobulin

**Fig. 1:**
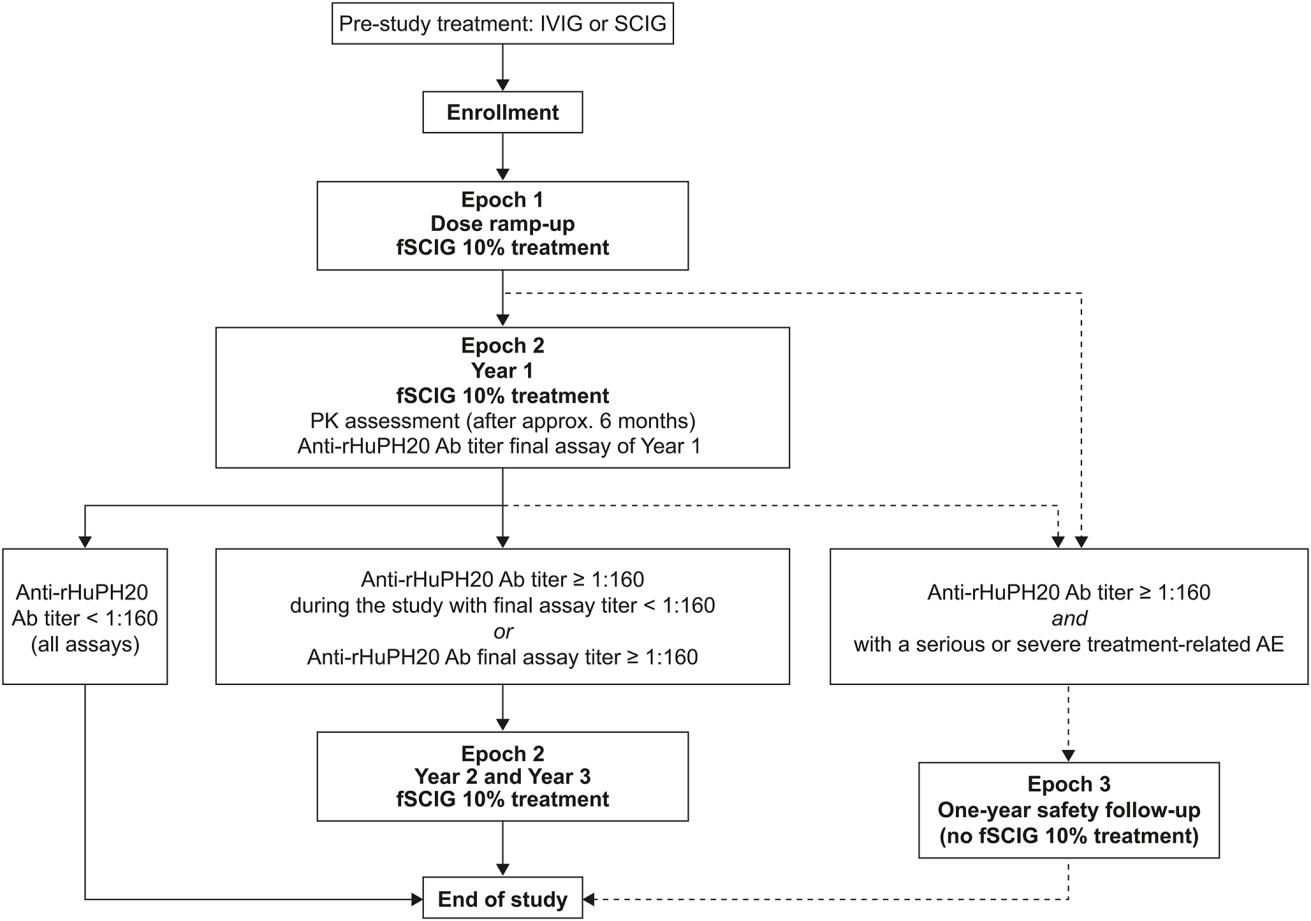
Study design. Epoch 1 (dose ramp-up): up to 6 weeks. Epoch 2 (final fSCIG 10% dosing): 1–3 years. Epoch 3 (safety follow-up): up to 1 year (at study completion, no participants entered Epoch 3). *Ab*, antibody; *AE*, adverse event; *fSCIG*, facilitated subcutaneous immunoglobulin; *IVIG*, intravenous immunoglobulin; *PK*, pharmacokinetics; *rHuPH20*, recombinant human hyaluronidase; *SCIG*, subcutaneous immunoglobulin

The study comprised three treatment epochs (Fig. 1). Participants were initially enrolled into Epoch 1 (dose ramp-up phase), in which they were treated with fSCIG 10% for up to 6 weeks. Participants with a body weight of < 40 kg received an initial dose of 5 mL/hour/site increasing to 80 mL/hour/site (maximum dose, if tolerated). Participants with a body weight of ≥ 40 kg received an initial dose of 10 mL/hour/site increasing to 240 mL/hour/site (maximum dose, if tolerated). All infusions during Epoch 1 were administered at the study site.

Epoch 1 was followed directly by Epoch 2, with the fSCIG 10% administration schedule dependent on how treatment was administered before enrollment (Fig. 1). Participants previously treated with IVIG received fSCIG 10% every 3 or 4 weeks depending on their previous IVIG dosing schedule (300–1000 mg/kg body weight/4 weeks); participants previously treated with SCIG received fSCIG 10% every 3 or 4 weeks at the discretion of the investigator and participant. After the initial infusions and training at the study site, for participants/caregivers deemed capable of self-infusion, home treatment was offered/recommended for subsequent infusions between pre-specified site visits throughout Epoch 2. Participants/caregivers were contacted by study sites 3–5 days after completion of each infusion throughout Epochs 1 and 2 to follow up on any AEs that may have occurred.

After 1 year of fSCIG 10% treatment in Epoch 2, results from anti-rHuPH20 binding antibody assays obtained during that year determined next steps. Participants with anti-rHuPH20 antibody titers < 1:160 for all assays conducted during the study completed the end-of-study visit at the next possible occasion after the 12-month visit. Participants with positive anti-rHuPH20 antibody titers of ≥ 1:160 (threshold chosen based on a previous study of anti-rHuPH20 antibodies in patients with PIDDs [16]) during the study and/or at the last measurement continued in Epoch 2 for an additional 2 years of fSCIG 10% treatment and observation. These participants completed the end-of-study visit at the next possible occasion after the 36-month visit.

An additional epoch, Epoch 3, was planned to allow for a further 1 year of safety follow-up (as required) with assessments every 3 months for participants with anti-rHuPH20 antibody titers ≥ 1:160 and a treatment-related serious or severe AE occurring during Epochs 1 or 2. However, no participants met these criteria, thus Epoch 3 was not employed in this study.

### Outcome Measures

The primary outcome was efficacy of fSCIG 10% as measured by the rate of acute serious bacterial infections (ASBIs), defined as the mean number of ASBIs per participant-year in the full analysis set (FAS) population. Secondary outcome measures further assessed efficacy (including the number of all infections per participant-year and serum IgG trough levels [during Epoch 2]), in addition to treatment characteristics, pharmacokinetics (PK) parameters (this outcome will be published separately), safety and tolerability, health-related quality of life (HRQoL), treatment preference, and healthcare resource utilization. A full list of all study outcomes and AE definitions (including definitions of ASBIs and AE seriousness, severity and causality) are provided in **Supplementary Methods**.

### Statistical Analysis

#### Sample Size

An estimate of 40 participants was deemed sufficient for enrollment to ensure a final sample size of 35 based on a dropout rate of 12% (determined by previous clinical experience). A sample size of 35 provides 83% power to reject the null hypothesis of an ASBI rate greater or equal to 1.0, by means of a one-sided test and a significance level of 0.01, versus the alternative hypothesis of less than 1.0, assuming a true rate of 0.5. A serious infection rate per person-year less than 1.0 is considered by the US Food and Drug Administration to be adequate to provide substantial evidence of efficacy [17].

#### Analysis Populations

The FAS included all participants who met study eligibility criteria and were enrolled into the study, and was used for all efficacy analyses. The safety analysis set included all participants who received at least one dose of fSCIG 10% and was used for all safety analyses.

#### Outcome Measures

The number and proportion of participants with any ASBI and the number of ASBIs were analyzed using descriptive statistics. Rates of events per participant-year were computed using a Poisson model and presented as point estimate and 95% confidence interval (CI). Total IgG trough levels, infection endpoints, safety, HRQoL, treatment preference/satisfaction, and healthcare resource utilization were analyzed using descriptive statistics and reported as proportions and rates.

## Results

### Participants

**Fig. 2:**
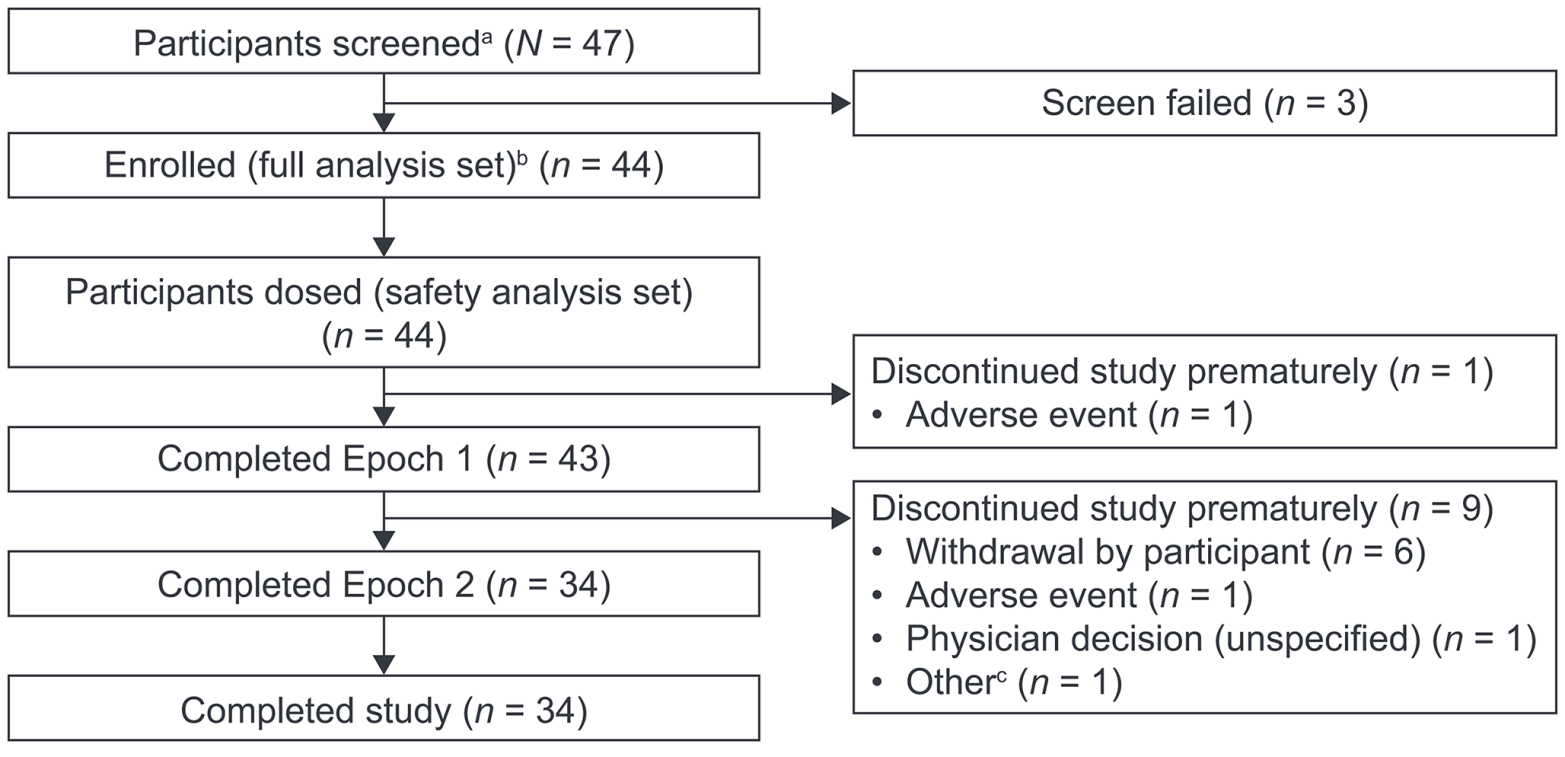
Participant disposition. ^a^All participants who provided informed consent. ^b^All participants who provided informed consent and met enrollment eligibility. ^c^A COVID-19-related constraint at the study site resulted in the participant being unable to complete the final study visit

Overall, 44 participants were enrolled; 34 participants completed the study with 10 participants prematurely discontinuing (Fig. 2). The overall mean (range) study duration was 14.1 (0.7–38.7) months.

In total, 43 participants completed Epoch 1 and entered Epoch 2; one participant prematurely discontinued Epoch 1 owing to an AE (worsening of pre-existing celiac disease). Of the 43 participants who entered Epoch 2, nine prematurely discontinued owing to withdrawal by the participant (*n* = 6; participants were free to withdraw from the study at any time without obligation to provide a reason for their decision), an AE (*n =* 1; infusion site pain), physician decision (*n =* 1), and for reasons related to COVID-19 (*n =* 1) (Fig. 2).

Baseline characteristics of the study population are summarized in Table 2. Mean ± standard deviation (SD) (median [range]) age of the study population was 9.0 ± 3.6 (9.5 [3–15]) years, with most participants (*n =* 23; 52.3%) in the 6 to < 12-year age group. Common variable immunodeficiency was the most common PIDD diagnosis at baseline (40.9%), followed by specific antibody deficiency (36.4%). Specific antibody deficiency was the most common diagnosis among participants aged 2 to < 6 years (77.8%), whereas common variable immunodeficiency was the most frequently reported diagnosis for those aged 6 to < 12 years (47.8%) and 12 to < 16 years (50.0%).

**Table 2:** Baseline characteristics

Characteristic	Age group, years			Overall (*N =* 44)
	2 to < 6 (*n =* 9)	6 to < 12 (*n =* 23)	12 to < 16 (*n =* 12)	
Age, years				
Mean ± SD	3.8 ± 0.7	8.7 ± 1.6	13.5 ± 1.2	9.0 ± 3.6
Median (range)	4 (3–5)	9 (6–11)	13.5 (12–15)	9.5 (3–15)
Sex, *n* (%)				
Male	5 (55.6)	13 (56.5)	8 (66.7)	26 (59.1)
Female	4 (44.4)	10 (43.5)	4 (33.3)	18 (40.9)
Race, *n* (%)				
Black or African American	1 (11.1)	0	1 (8.3)	2 (4.5)
White	6 (66.7)	23 (100)	11 (91.7)	40 (90.9)
Other	1 (11.1)	0	0	1 (2.3)
Multiple	1 (11.1)	0	0	1 (2.3)
Ethnicity, *n* (%)				
Hispanic or Latino	3 (33.3)	2 (8.7)	0	5 (11.4)
Not Hispanic or Latino	6 (66.7)	21 (91.3)	12 (100)	39 (88.6)
Height, cm				
Mean ± SD	97.6 ± 7.8	134.0 ± 12.8	159.9 ± 8.3	133.6 ± 24.0
Median (range)	95.8 (86.1–106.7)	133.4 (112.8–157.0)	159.3 (146.6–170.2)	137.8 (86.1–170.2)
Weight, kg				
Mean ± SD	15.4 ± 2.6	34.6 ± 12.7	60.7 ± 14.6	37.8 ± 19.9
Median (range)	14.8 (11.9–18.8)	31.1 (19.1–64.6)	59.5 (42.6–92.7)	34.5 (11.9–92.7)
PIDD diagnosis, *n* (%)				
Agammaglobulinemia^a^	0	3 (13.0)	0	3 (6.8)
CVID	1 (11.1)	11 (47.8)	6 (50.0)	18 (40.9)
Severe combined immunodeficiency	1 (11.1)	2 (8.7)	0	3 (6.8)
Specific antibody deficiency^b^	7 (77.8)	6 (26.1)	3 (25.0)	16 (36.4)
Other^c^	0	1 (4.3)	3 (25.0)	4 (9.1)
Medical history, *n* (%)^d^				
Aphthous ulcer	–	–	–	3 (6.8)
Irritable bowel syndrome	–	–	–	3 (6.8)
Fatigue	–	–	–	3 (6.8)
Otitis media chronic	–	–	–	3 (6.8)
Arthralgia	–	–	–	3 (6.8)
Speech disorder development	–	–	–	3 (6.8)
Dermatitis atopic	–	–	–	3 (6.8)
Urticaria	–	–	–	3 (6.8)
Sinus operation	–	–	–	3 (6.8)
Combined immunodeficiency	–	–	–	4 (9.1)
Upper respiratory tract infection	–	–	–	4 (9.1)
Influenza	–	–	–	4 (9.1)
Molluscum contagiosm	–	–	–	4 (9.1)
Respiratory tract infection	–	–	–	4 (9.1)
Adenotonsillectomy	–	–	–	4 (9.1)
Abdominal pain	–	–	–	5 (11.4)
Bronchitis	–	–	–	5 (11.4)
Myringotomy	–	–	–	5 (11.4)
Insomnia	–	–	–	5 (11.4)
Constipation	–	–	–	6 (13.6)
Anxiety	–	–	–	6 (13.6)
Eczema	–	–	–	6 (13.6)
Seasonal allergy	–	–	–	7 (15.9)
Ear infection	–	–	–	7 (15.9)
Sinusitis	–	–	–	7 (15.9)
Food allergy	–	–	–	8 (18.2)
Drug hypersensitivity	–	–	–	9 (20.5)
Chronic sinusitis	–	–	–	9 (20.5)
Rhinitis allergic	–	–	–	9 (20.5)
Hypogammaglobulinaemia	–	–	–	10 (22.7)
Otitis media	–	–	–	10 (22.7)
Headache	–	–	–	10 (22.7)
Attention deficit hyperactivity disorder	–	–	–	10 (22.7)
Immunodeficiency common variable	–	–	–	11 (25.0)
Rhinitis	–	–	–	11 (25.0)
Tonsillectomy	–	–	–	13 (29.5)
Gastroesophageal reflux disease	–	–	–	14 (31.8)
Pneumonia	–	–	–	14 (31.8)
Adenoidectomy	–	–	–	16 (36.4)
Ear tube insertion	–	–	–	16 (36.4)
Asthma	–	–	–	30 (68.2)

^a^Includes X-linked agammaglobulinemia (Bruton’s agammaglobulinemia) and autosomal recessive agammaglobulinemia/hypogammaglobulinemia

^b^Includes specific antibody deficiency with hypogammaglobulinemia/low IgG, other specific antibody deficiency with IgG subclass deficiency, and other specific antibody deficiency

^c^Includes two participants with “IgG subclass deficiency, low IgA”, one participant with “hypogammaglobulinemia”, and one participant with “hypogammaglobulinemia and IgG subclass deficiency”

^d^Medical history by preferred term occurring in > 5% of participants

*CVID*, common variable immunodeficiency; *IgA*, immunoglobulin A; *IgG*, immunoglobulin G; *PIDD*, primary immunodeficiency disease; *SD*, standard deviation

### Acute Serious Bacterial Infections (ASBIs)

Two ASBIs, both cases of bacterial pneumonia, occurred in one participant (2.3%) with a diagnosis of specific antibody deficiency (normal immunoglobulin concentration and number of B cells). The first ASBI occurred during Epoch 1 and the second during Epoch 2; both events were not temporally associated with the last infusion received, occurring after more than 72 h from the last infusion. Past medical history for the participant included bronchiolitis, respiratory syncytial virus infection, gastroesophageal reflux disease, and tracheomalacia.

The mean rate of ASBIs was 0.04 per participant-year (99% upper CI: 0.20), significantly (*p <* 0.001) lower than the regulatory-defined threshold rate of 1.0 [17]. Therefore, the primary endpoint of the study was met.

### All Infections

Overall, 161 infections were experienced by 34 participants (77.3%), equivalent to a mean rate of 3.12 events per participant-year (95% upper CI limit: 3.95). Mean infection rates per participant-year by age group were 4.01, 3.23, and 1.97 for the 2 to < 6, 6 to < 12, and 12 to < 16-year age groups, respectively. All infections occurring in at least one participant with an infectious event are reported in Supplementary Table S1. The most commonly reported infections (occurring in > 10% of participants) by preferred term across the entire study were as follows: sinusitis (including preferred terms for sinusitis and acute sinusitis: 32 events in 23 participants [52.3%]), upper respiratory tract infection (URTI; including preferred terms for URTI and viral URTI: 24 events in 18 participants [40.9%]), streptococcal pharyngitis (11 events in 7 participants [15.9%]), influenza (6 events in 6 participants [13.6%]), and otitis media (including preferred terms for acute otitis media and chronic otitis media: 16 events in 8 participants [18.2%]).

### Treatment Characteristics

The overall mean ± SD duration of exposure to fSCIG 10% during the study was 14.0 ± 5.4 months (Epoch 1: 1.3 ± 0.3 months; Epoch 2: 13.0 ± 5.1 months).

In Epoch 2, 633 infusions were administered (145, 339, and 149 infusions in the 2 to < 6, 6 to < 12, and 12 to < 16-year age groups, respectively). Treatment characteristics for infusions administered during Epoch 2 are summarized in Table 3. Across all age groups, participants received a median (range) of 1.1 (1.0–1.5) infusions per month. The median (range) number of infusions per participant-year was 13.2 (11.5–17.7). Overall, the median (range) number of infusion sites per month was 2.2 (1.1–2.9), with similar median values across age groups. The mean ± SD number of infusion sites per infusion was 1.8 ± 0.4 and the median (range) was 2.0 (1.0–2.0), which were comparable across age groups. The overall median (range) duration of infusion was 85 (45–215) minutes. The median (range) infusion volume was 85 (35–300) mL/site, and was lowest in the 2 to < 6-year age group (60.0 mL/site) and highest in the 12 to < 16-year age group (142.5 mL/site), with a maximum (range) infusion rate of 160 (30–300) mL/hour/site. The first 2–3 infusions during Epoch 2 were mandatorily administered at study sites; thereafter 74.4% of participants received ≥ 2 infusions at home and 45.3% of all infusions during Epoch 2 were administered in the home setting.

**Table 3:** Treatment characteristics for fSCIG 10% in Epoch 2

	Age group, years			Overall (*N =* 43)
	2 to < 6 (*n =* 9)	6 to < 12 (*n =* 22)	12 to < 16 (*n =* 12)	
Number of infusions per month	1.1 (1.0–1.3)	1.1 (1.0–1.4)	1.1 (1.0–1.5)	1.1 (1.0–1.5)
Number of infusions per participant-year	13.0 (12.2–15.0)	13.2 (12.1–17.4)	13.2 (11.5–17.7)	13.2 (11.5–17.7)
Infusion volume/site, mL	60 (35–143)	85 (60–178)	143 (75–300)	85 (35–300)
Infusion duration, minutes	77 (60–215)	88 (53–150)	80 (45–163)	84 (45–215)
Number of infusion sites per month	2.0 (1.1–2.2)	2.2 (1.1–2.9)	2.2 (1.4–2.9)	2.2 (1.1–2.9)
Maximum infusion rate/site, mL/hour	160 (40–160)	160 (30–300)	300 (30–300)	160 (30–300)
Participants with infusions interrupted owing to an AE, *n* (%)	3 (33.3)	6 (27.3)	4 (33.3)	13 (30.2)
Dosing interval, *n* (%)^a^				
Every 3 weeks	1 (11.1)	4 (18.2)	3 (25.0)	8 (18.6)
Every 4 weeks	9 (100)	18 (81.8)	9 (75.0)	36 (83.7)

Data shown are median (range) unless otherwise stated

^a^Percentages may sum to more than 100% because assigned treatment interval could be changed during the study

### Serum IgG Trough Levels

**Fig. 3:**
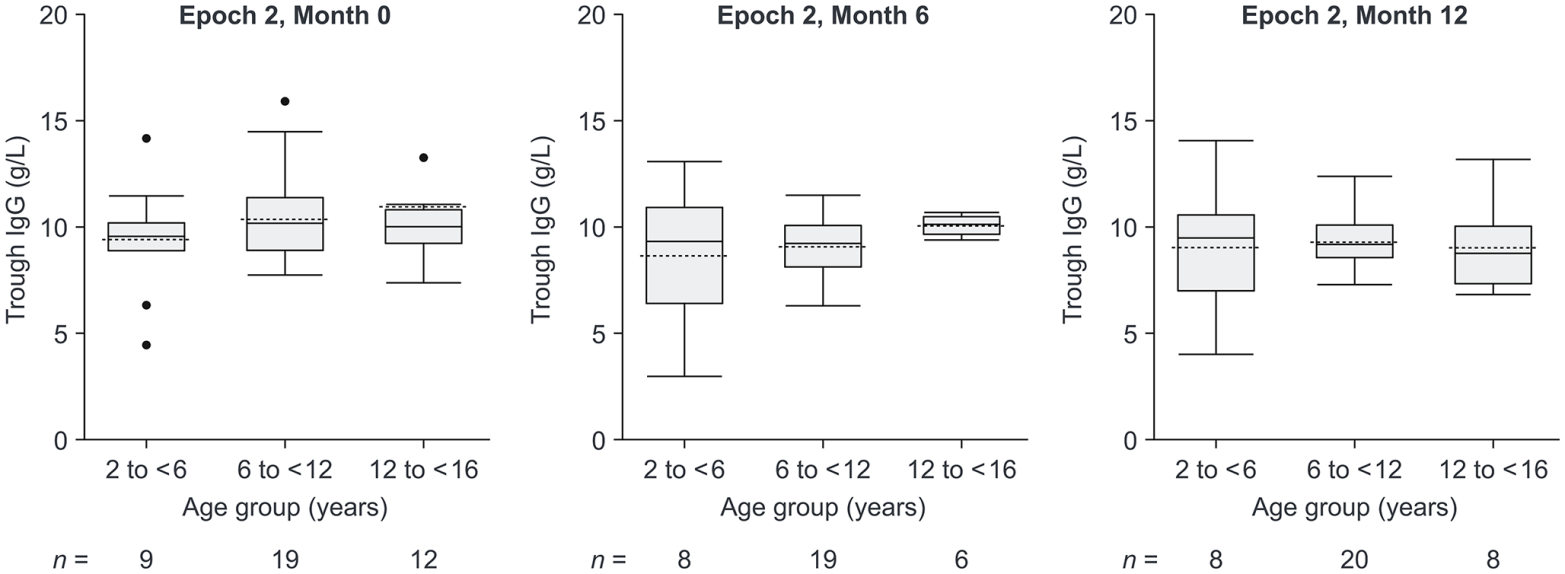
Box plots of serum trough levels of total IgG by visit and age group. Age group is based on participant age at screening. The length of a box represents the IQR. The dashed line in the box indicates the group mean, and the horizontal line reflects the group median. The vertical lines issuing from the box extend to the group minimum (Q1 − 1.5 × IQR) and maximum (Q3 + 1.5 × IQR) values. Outliers are shown as circles. *IgG*, immunoglobulin G; *IQR*, interquartile range; *Q*, quartile

Mean ± SD (median [range]) serum total IgG levels at baseline were similar between participants previously receiving IVIG and SCIG (9.8 ± 1.9 [10.5 (6.1–11.1)] and 10.1 ± 3.2 [9.3 (4.6–24.4)] g/L, respectively). For the overall study population, mean ± SD (median [range]) serum IgG trough levels were 10.38 ± 2.91 (10.05 [4.46–22.90]), 9.20 ± 1.96 (9.42 [3.04–13.10]), and 9.21 ± 1.98 (9.29 [4.02–14.10]) g/L at Months 0, 6, and 12, respectively, of Epoch 2. IgG trough levels stayed stable between visits (Table 4). In addition, serum IgG trough levels were generally similar across age groups, although participants in the 2 to < 6-year age group had slightly lower levels compared with other age groups. Additionally, mean serum total IgG concentration profiles were similar for each age group (Fig. 3).

**Table 4:** IgG trough levels during Epoch 2 in the full analysis set

	Age group, years			Overall (*N =* 44)
	2 to < 6 (*n =* 9)	6 to < 12 (*n =* 23)	12 to < 16 (*n =* 12)	
Month 0				
* n*	*9*	*19*	*12*	*40*
Mean ± SD, g/L	9.43 ± 2.80	10.43 ± 2.10	11.01 ± 4.02	10.38 ± 2.91
Median (range), g/L	9.58 (4.46–14.20)	10.20 (7.77–15.90)	10.04 (7.40–22.90)	10.05 (4.46–22.90)
Month 6				
* n*	*8*	*19*	*6*	*33*
Mean ± SD, g/L	8.70 ± 3.30	9.12 ± 1.45	10.10 ± 0.51	9.20 ± 1.96
Median (range)	9.34 (3.04–13.10)	9.25 (6.29–11.50)	10.15 (9.42–10.70)	9.42 (3.04–13.10)
Month 12				
* n*	*8*	*20*	*8*	*36*
Mean ± SD, g/L	9.04 ± 3.15	9.35 ± 1.37	9.05 ± 2.11	9.21 ± 1.98
Median (range)	9.49 (4.02–14.10)	9.21 (7.31–12.40)	8.78 (6.82–13.20)	9.29 (4.02–14.10)

*IgG*, immunoglobulin; *SD*, standard deviation

### Safety and Tolerability

#### Adverse Events

Excluding infections, 536 treatment-emergent AEs (TEAEs) were recorded by 43 participants, of which 336 events in 34 participants were considered treatment-related with the vast majority of these mild in nature (Table 5). The most common TEAEs (excluding infections) were infusion/injection site pain (52.3% of participants), headache (43.2%), and infusion/injection site pruritus (29.5%) (Table 6). The overall rate of treatment-related TEAEs (excluding infections) was 0.443 events per infusion. This rate decreased over time from Epoch 1 (0.675 events/infusion) to Epoch 2 (0.397 events/infusion) (Table 5). Most treatment-related TEAEs were mild (247 events [73.5% of related TEAEs]) or moderate (87 events [25.9%]) in severity, and were reported in 32 participants (72.7%) and 19 participants (43.2%), respectively. Three severe AEs (excluding infections), all of which were systemic, were reported in three participants; two of the severe AEs were assessed as treatment-related (celiac disease flare and headache). One serious AE (excluding infections) was reported (tonsillar hypertrophy) but was not considered to be treatment-related. The overall rate of treatment-related systemic and local TEAEs (excluding infections) were 0.190 and 0.253 events per infusion, respectively, and decreased over time from Epoch 1 (systemic, 0.214; local, 0.460) to Epoch 2 (systemic, 0.185; local, 0.212) (Table 5). A summary of TEAEs including infections is presented in Supplementary Table S2.

**Table 5:** Summary of TEAEs, excluding infections, by epoch and overall in the safety analysis set

	Epoch 1 (*n =* 44)			Epoch 2 (*n =* 43)			Overall (*N =* 44)		
	Participants, *n* (%)	Events, *n*	Events per infusion	Participants, *n* (%)	Events, *n*	Events per infusion	Participants, *n* (%)	Events, *n*	Events per infusion
TEAEs	29 (65.9)	119	0.944	40 (93.0)	417	0.659	43 (97.7)	536	0.706
Related	25 (56.8)	85	0.675	31 (72.1)	251	0.397	34 (77.3)	336	0.443
Serious TEAEs	0	0	0	1 (2.3)	1	0.002	1 (2.3)	1	0.001
Related	0	0	0	0	0	0	0	0	0
Severe TEAEs	>1 (2.3)	1	0.008	2 (4.7)	2	0.003	3 (6.8)	3	0.004
Related	1 (2.3)	1	0.008	1 (2.3)	1	0.002	2 (4.5)	2	0.003
Moderate TEAEs	14 (31.8)	25	0.198	27 (62.8)	116	0.183	31 (70.5)	141	0.186
Related	11 (25.0)	20	0.159	14 (32.6)	67	0.106	19 (43.2)	87	0.115
Mild TEAEs	26 (59.1)	93	0.738	40 (93.0)	299	0.472	43 (97.7)	392	0.516
Related	23 (52.3)	64	0.508	28 (65.1)	183	0.289	32 (72.7)	247	0.325
Local TEAEs	22 (50.0)	60	0.476	29 (67.4)	142	0.224	33 (75.0)	202	0.266
Related	22 (50.0)	58	0.460	28 (65.1)	134	0.212	32 (72.7)	192	0.253
Systemic TEAEs	23 (52.3)	59	0.468	37 (86.0)	275	0.434	39 (88.6)	334	0.440
Related	12 (27.3)	27	0.214	20 (46.5)	117	0.185	25 (56.8)	144	0.190

*TEAE*, treatment-emergent adverse event

**Table 6:** Summary of the most common TEAEs, excluding infections (occurring in ≥ 10% of participants) in Epochs 1 and 2 combined (*N* = 44 participants)

System organ class Preferred term	Participants, *n* (%)	Events, *n*	Events per infusion
Gastrointestinal disorders			
Vomiting	10 (22.7)	12	0.016
Diarrhea	9 (20.5)	10	0.013
Nausea	7 (15.9)	9	0.012
General disorders and administration site conditions			
Infusion/injection site pain	23 (52.3)	61	0.080
Infusion/injection site pruritus	13 (29.5)	24	0.032
Infusion/injection site erythema	12 (27.3)	39	0.051
Pyrexia	12 (27.3)	18	0.024
Infusion/injection site extravasation	11 (25.0)	22	0.029
Infusion/injection site swelling	10 (22.7)	23	0.030
Fatigue	7 (15.9)	10	0.013
Infusion/injection site discoloration	5 (11.4)	10	0.013
Injury, poisoning, and procedural complications			
Infusion-related reaction	6 (13.6)	8	0.011
Nervous system disorders Headache	19 (43.2)	84	0.111
Respiratory, thoracic, and mediastinal disorders			
Asthma	5 (11.4)	12	0.016
Epistaxis	5 (11.4)	10	0.013
Skin and subcutaneous tissue disorders			
Pruritus	5 (11.4)	6	0.008
Rash	5 (11.4)	7	0.009

Safety analysis set

*TEAE*, treatment-emergent adverse event

**Fig. 4:**
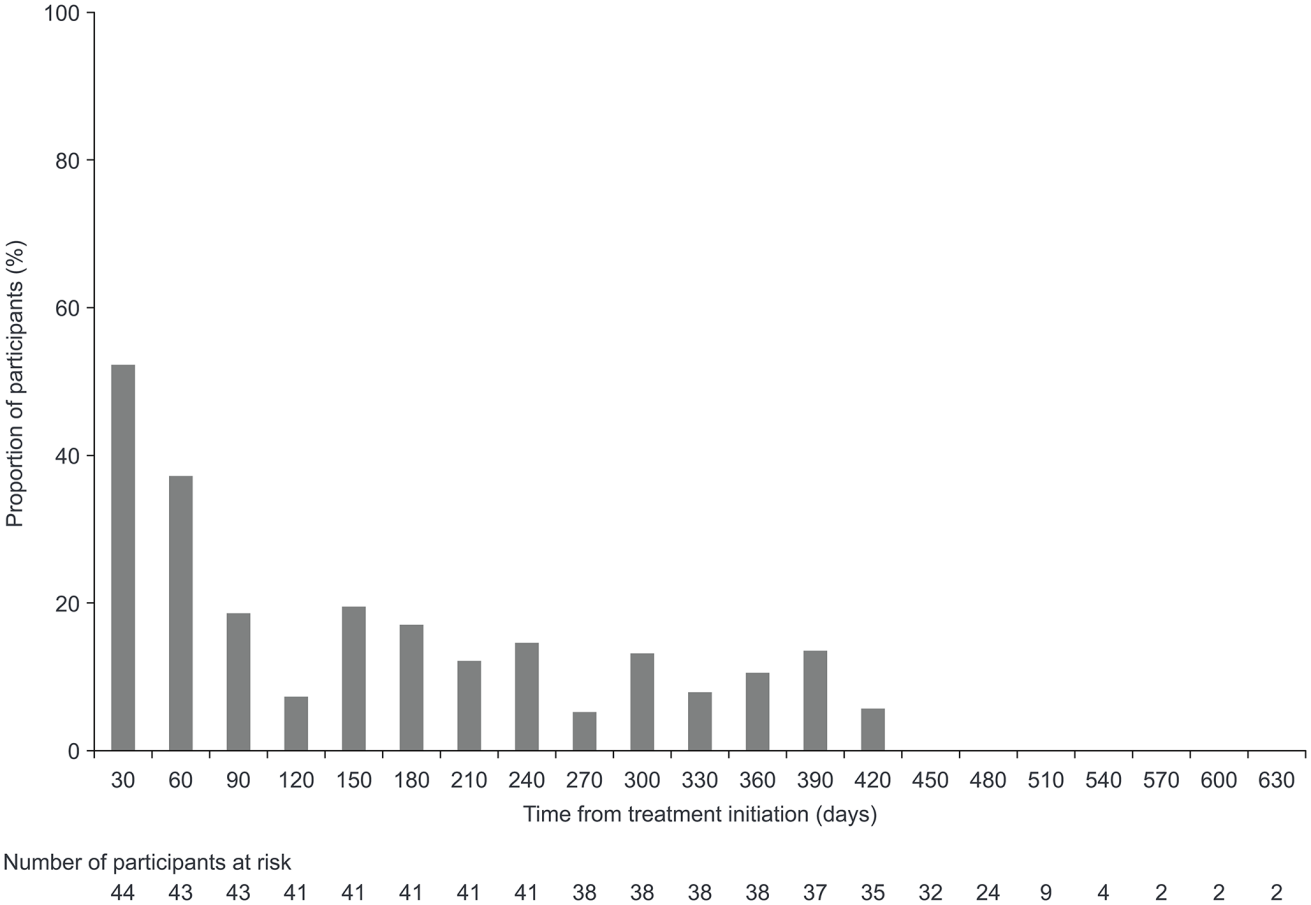
Frequency of local TEAEs over time. The x axis represents time intervals for each month (30 days). The first bar is the proportion of participants with a TEAE that started between Day 1 and Day 30, the second bar is the proportion of participants with a TEAE that started between Day 31 and Day 60, and so on. The proportion was computed as follows: 100 × (number of participants with an event/number of participants at risk for an event in that time period). One participant was observed for up to 1200 days and experienced two TEAEs (not shown). *TEAE*, treatment-emergent adverse event

The overall rates of any TEAEs (excluding infections) per infusion and per participant were 0.706 and 12.182, respectively, equivalent to a rate of 10.375 events per participant-year. These rates declined over time from Epoch 1 to Epoch 2. Thus, in Epoch 1, the equivalent rates were 0.944 and 2.705 with a rate of 25.719 events per participant-year. For Epoch 2, the corresponding rates were 0.659 and 9.698 with a rate of 8.865 events per participant-year. The proportion of participants with any local TEAE was also highest at the start of treatment and decreased rapidly over the first 120 days and had further decreased by Day 450 (Fig. 4). After Day 630, only one participant was still receiving treatment; the participant was observed for up to 1200 days and reported two TEAEs during this time.

Overall, 13 participants (30.2%) had 17 infusions that were interrupted owing to an AE (2.7% of infusions administered in Epoch 2; Table 3), and the proportion of patients with interruptions was broadly similar across the three age groups evaluated (33.3%, 27.3%, and 33.3% in the 2 to < 6, 6 to < 12, and 12 to < 16-year age groups, respectively). AEs that contributed to interruption of infusion (alone or in combination) included infusion site extravasation, erythema, swelling, pain and pruritus, and dizziness. Of all infusions administered over the course of the study, 99.8% were completed; 94.1% were completed without interruption.

#### Binding and Neutralizing Antibodies against rHuPH20

One participant with specific antibody deficiency developed an anti-rHuPH20 binding antibody titer ≥ 1:160 rHuPH20 at Month 6 of Epoch 2 (titer, 1:640). At Months 9, 12, and 15 of Epoch 2, anti-rHuPH20 binding antibody titers were 1:1280, 1:2560, and 1:2560, respectively, followed by titers of 1:10,240 (Month 18), 1:2560 (Month 21), 1:5120 (Month 24), and 1:10,240 (Month 27). Titers then decreased to 1:2560 (Month 30, unscheduled visit) and were at 1:5120 by the end of the study (Epoch 2, Month 36). The participant experienced local and systemic TEAEs (mostly headache) before and after developing anti-rHuPH20 binding antibodies. All treatment-related TEAEs in this participant fully resolved without sequelae and none were considered serious or severe, or considered to be a result of an immune-mediated response. Additional characterization of antibodies in this participant determined that they did not contain antibodies cross-reacting with Hyal 1, 2, and 4, and there was no evidence of complement consumption or immune complex formation. Total IgG trough levels for this participant remained stable throughout Epoch 2, and there was no association between maximum IgG concentration and rHuPH20-reactive antibodies. The overall incidence of treatment-emergent anti-rHuPH20 binding antibodies was 2.3% (1/44 participants). No rHuPH20 neutralizing antibodies were detected in any participant.

### Participant-Reported Outcomes

#### Health-Related Quality of Life

At the end of Epoch 2, mean ± SD changes from baseline in the Pediatric Quality of Life Inventory (PedsQL) total score were small in magnitude and similar across age groups: 8.2 ± 17.7, − 4.6 ± 16.3, − 2.0 ± 14.0, and − 2.5 ± 8.8 for participants in the 2–4, 5–7, 8–12, and 13–< 16-year age groups, respectively. Given the small sample size in each group, these data should be interpreted with caution.

#### Treatment Preference

At the end of Epoch 2, positive responses were recorded by most participants who completed the treatment preference questionnaire (*n =* 42). The highest-rated aspects of treatment were frequency of administration and overall convenience; 88% and 81% of respondents, respectively, rated these aspects as “liked very much” or “liked”. Most participants also expressed a preference for continuing fSCIG 10% (74%) and for receiving treatment at home (86%).

### Healthcare Resource Utilization

The median (range) number of days wherein a participant was not able to go to school/work or to perform normal daily activities due to infection or other illness was 1.5 (0–51) days per participant. This equated to a mean rate of 4.28 (standard error [SE]: 1.00) (95% CI: 2.71–6.75) days per participant-year.

In total, 28 participants (63.6%) received antibiotics during the study, with a median (range) per participant of 3.0 (1.0–11.0) courses and 34.5 (5.0–343.0) days on treatment during the study. This equated to a mean (SE) rate per participant-year of 2.2 (0.4) antibiotic courses and 26.8 (5.5) days on antibiotic treatment.

Four participants were hospitalized. The mean rate of infection-related hospitalizations was 0.06 (SE: 0.03) (95% CI: 0.02–0.18) per participant-year; the median (range) number of infection-related hospitalizations per participant was 0.0 (0.0–1.0). The median (range) number of days hospitalized per participant was 0.0 (0.0–4.0), with a mean rate of 0.21 (SE: 0.07) (95% CI: 0.11–0.42) days hospitalized per participant-year.

## Discussion

This phase 3 study, conducted in US pediatric participants with PIDDs, met its primary endpoint, demonstrating the efficacy of fSCIG 10% in preventing bacterial infection in this population. The mean ASBI rate per participant-year (0.04) was significantly lower than the regulatory-defined threshold rate of 1.00 (*p <* 0.001) [17]. No clinically meaningful differences in IgG trough levels were observed and total IgG exposure was similar among pediatric age groups, with stable and protective IgG levels maintained throughout the study. The decrease in mean IgG trough levels observed in the overall study population between Month 0 and Month 12 of Epoch 2 (decrease < 1.3 g/L) was not clinically significant, and may be explained in part by the pooling of participants who had received different IgRT regimens before receiving study treatment of fSCIG 10%. For example, weekly dosing of SCIG is expected to result in a higher IgG trough level with less fluctuation before and after dosing than monthly IVIG dosing [18].

Only one participant developed anti-rHuPH20 binding antibodies during the course of the study; this participant continued fSCIG 10% with no change to their treatment regimen and did not develop rHuPH20 neutralizing antibodies at any point. Furthermore, AEs experienced by this participant were mild, transient, and not considered to be a result of an immune-mediated response. Furthermore, administration parameters and IgG trough levels remained stable, suggesting that anti-rHuPH20 binding antibodies did not have a functional effect. The overall incidence of treatment-emergent anti-rHuPH20 antibodies in the current study was 2.3%, which was substantially lower than that observed in the pivotal phase 3 trial of fSCIG 10% in a mixed adult and pediatric population with PIDDs (18.1%) [14].

The current study supports the tolerability of fSCIG 10%, showing a similar infusion duration to that observed in the pooled analysis of pediatric patients in the pivotal phase 3 trial and its extension study [13]. Headache was a frequently reported TEAE in the current study (43.2% of participants). Although this is a higher frequency than typically seen with conventional SCIG, it is consistent with other studies of fSCIG 10% [13, 14, 19]; however it is worth noting that rate of headaches per infusion may be a more clinically useful measure. The current study also reports a median of 2.2 infusion sites per month and an overall median infusion duration of 85 min in this analysis compared with 1.09 sites per month and 99 min in the prior study [13]. fSCIG 10% tolerability is also demonstrated by the low rate of local AEs after 120 days through to the end of the study. Furthermore, fSCIG 10% can be administered at home, with almost half of all infusions in the current study administered in a home setting. A retrospective chart analysis of pediatric fSCIG 10% usage conducted at three centers in Germany further supports the feasibility and tolerability of administering fSCIG 10% to pediatric participants with immunodeficiencies at home every 3–4 weeks [20]. Efficacy, safety, and tolerability were comparable with other modes of immunoglobulin administration. In addition, the reduction in the number of infusions and needle sticks per month for fSCIG 10%, combined with self/parental administration at home, is a potentially important advantage over conventional SCIG.

fSCIG 10% was administered at the same dosing interval as the participants’ pre-study IVIG treatment for PIDDs (3- or 4-week treatment intervals), and was the preferred mode of treatment for the vast majority of participants. When participants were asked to rate different aspects of treatment, frequency of administration and overall convenience were the highest-rated aspects of fSCIG 10% treatment, with the ability to infuse at home also rated highly by participants. In conjunction with this, PedsQL scores remained stable throughout the study, demonstrating only small changes in HRQoL from baseline to the end of Epoch 2.

Overall, the safety profile of fSCIG 10% in this pediatric population was favorable and consistent with observations made in pediatric participants with PIDDs in other clinical trials [13, 14]. Indeed, the rate of ASBIs observed in the current study (0.04 per participant-year) was similar to (and somewhat lower than) that reported in pediatric patients participating in the pivotal phase 3 study of fSCIG 10% where a rate of 0.08 events per participant-year was reported [13]. Furthermore, the rate of AEs per infusion decreased over time in the current study, most notably within the first 120–150 days, with fewer events per infusion, per participant, and per participant-year in Epoch 2 than in Epoch 1. Subcutaneous administration of immunoglobulin is associated with lower rates of systemic AEs than IVIG [21], and rates of treatment-related systemic TEAEs (excluding infections) per infusion decreased from Epoch 1 to Epoch 2 in the current study. Overall decline in the rates of TEAEs may be related to an improvement in participants’ tolerance over time.

Strengths of this study include participation of patients from multiple centers across the USA, with a good study completion rate and the lengthy follow-up period of > 3 years over the entire study, resulting in a substantial duration of fSCIG 10% exposure.

Limitations of the study should also be acknowledged. Due to the small number of participants, diagnoses of specific antibody deficiency may not be representative of the general patient population; however, all participants had PIDD according to the International Union of Immunological Societies criteria and required immunoglobulin treatment. In general, the incidence of AEs may be under-reported because of the varying reliability of reporting by young children manifesting symptoms. However, the close parental/caregiver observation during and after infusions mitigates this possibility. In addition, assessment of participant-reported outcomes, including HRQoL, is limited by small sample sizes in the individual age groups assessed and by variability in scores. Last, the evolving COVID-19 pandemic during the study caused the interruption of scheduled visits, resulting in some protocol deviations and the discontinuation of one participant (due to a COVID-19-related constraint at the study site). However, the protocol deviations did not affect key efficacy or safety assessments.

## Conclusions

This study of US pediatric participants with PIDDs (also referred to as inborn errors of immunity) demonstrated that fSCIG 10% was effective in preventing infections with a safety profile consistent with that observed in previous studies. In addition, consistent with previous clinical studies, stable and protective IgG levels were maintained across all pediatric age groups evaluated. fSCIG 10% was administered at the same dosing interval as IVIG therapies for PIDDs, and potentially offers a more convenient treatment option that is better suited for home treatment.

## Electronic Supplementary Material

Below is the link to the electronic supplementary material.

Supplementary Material 1

Supplementary Material 2

Supplementary Material 3

## Data Availability

The study is registered with the ClinicalTrials.gov registry at https://clinicaltrials.gov/ct2/show/NCT03277313 (ClinicalTrials.gov Identifier: NCT03277313). Information pertaining to this study and the associated study protocol may be found at ClinicalTrials.gov. The data sets, including the redacted study protocol, redacted statistical analysis plan, and individual participants’ data supporting the results reported in this article, will be made available within three months from initial request to researchers who provide a methodologically sound proposal. The data will be provided after its de-identification, in compliance with applicable privacy laws, data protection, and requirements for consent and anonymization.
