# Supplementary material for: Efficacy, Safety, Tolerability, and Serum IgG Trough Levels of Hyaluronidase-Facilitated Subcutaneous Immunoglobulin 10% in US Pediatric Patients with Primary Immunodeficiency Diseases
Source: J Clin Immunol. 2025 Mar 14;45(1):81. doi: 10.1007/s10875-025-01862-6 (PMC11909037; doi:10.1007/s10875-025-01862-6)
Supplement: Supplementary file 1 — Supplementary Material 1 [file 10875_2025_1862_MOESM1_ESM.pdf]

Efficacy, Safety, Tolerability, and Serum IgG Trough Levels of Hyaluronidase-facilitated Subcutaneous Immunoglobulin 10% in US Pediatric Patients with Primary Immunodeficiency Diseases

Journal of Clinical Immunology

Niraj C. Patel • Jolan E. Walter • Richard L. Wasserman • Arye Rubinstein • Suthida Kankirawatana • Meagan W. Shepherd • Erin Greco • Zhaoyang Li • Sharon Russo-Schwarzbaum • Shumyla Saeed-Khawaja • Barbara McCoy • Leman Yel

Corresponding author:
Leman Yel, M.D.
lyel@uci.edu

Supplementary Results

Table S2 Summary of TEAEs, including infections, by epoch and overall, in the safety analysis set

	Epoch 1 (*N* = 44)		Epoch 2 (*N* = 43)		Overall (*N* = 44)	
	**Participants, *n* (%)**	**Events, *n***	**Participants, *n* (%)**	**Events, *n***	**Participants, *n* (%)**	**Events, *n***
TEAEs	31 (70.5)	142	40 (93.0)	555	43 (97.7)	697
Related	25 (56.8)	85	31 (72.1)	251	34 (77.3)	336
Serious TEAEs	1 (2.3)	1	3 (7.0)	3	4 (9.1)	4
Related	0	0	0	0	0	0
Severe TEAEs	1 (2.3)	1	3 (7.0)	3	4 (9.1)	4
Related	1 (2.3)	1	1 (2.3)	1	2 (4.5)	2
Local TEAEs	22 (50.0)	60	29 (67.4)	142	33 (75.0)	202
Related	22 (50.0)	58	28 (65.1)	134	32 (72.7)	192
Systemic TEAEs	29 (65.9)	82	38 (88.4)	413	41 (93.2)	495
Related	12 (27.3)	27	20 (46.5)	117	25 (56.8)	144

TEAE, treatment-emergent adverse event
