# Supplementary material for: Efficacy, Safety, Tolerability, and Serum IgG Trough Levels of Hyaluronidase-Facilitated Subcutaneous Immunoglobulin 10% in US Pediatric Patients with Primary Immunodeficiency Diseases
Source: J Clin Immunol. 2025 Mar 14;45(1):81. doi: 10.1007/s10875-025-01862-6 (PMC11909037; doi:10.1007/s10875-025-01862-6)
Supplement: Supplementary file 2 — Supplementary Material 2 [file 10875_2025_1862_MOESM2_ESM.pdf]

Efficacy, Safety, Tolerability, and Serum IgG Trough Levels of Hyaluronidase-facilitated Subcutaneous Immunoglobulin 10% in US Pediatric Patients with Primary Immunodeficiency Diseases

Journal of Clinical Immunology

Niraj C. Patel • Jolan E. Walter • Richard L. Wasserman • Arye Rubinstein • Suthida Kankirawatana • Meagan W. Shepherd • Erin Greco • Zhaoyang Li • Sharon Russo-Schwarzbaum • Shumyla Saeed-Khawaja • Barbara McCoy • Leman Yel

Corresponding author:
Leman Yel, M.D.
lyel@uci.edu

Supplementary Methods

Outcome Measures

Primary Efficacy Outcome Measure

- The rate of acute serious bacterial infections (ASBIs), defined as the mean number of ASBIs per participant-year in the intent-to-treat population (full analysis set).

Secondary Outcome Measures

Efficacy

- Number of all infections per participant-year.
- Trough levels of immunoglobulin G (IgG) and IgG subclasses for Epoch 2.
- Trough levels of specific antibodies to clinically relevant pathogens (*Clostridium tetani* toxoid, *Haemophilus influenzae,* and hepatitis B virus) for Epoch 2.

Pharmacokinetics (PK) (Epoch 2)

- For PK assessment in Epoch 2, the following PK parameters were determined for observed total IgG levels: area under the curve (AUC), apparent clearance (CL/F), maximum concentration (C_{max}), minimum concentration (C_{min}), time to maximum

concentration (T_{max}), and terminal half-life. Additionally, baseline-corrected total IgG AUC, C_{max}, and T_{max} were calculated.

Safety

- Number and rate per infusion (excluding infections) of serious adverse events, related and not related.
- Number and rate per infusion (excluding infections) of all adverse events (AEs), related and not related.
- Number and rate per infusion (excluding infections) of local AEs, related and not related.
- Number and rate per infusion (excluding infections) of systemic AEs, related and not related.
- Number and rate per infusion (excluding infections) of all temporally associated AEs (starting during or within 72 hours after completion of infusion).
- Number and rate per infusion (excluding infections) of all causally related and/or temporally associated AEs.
- Rates of all AEs (excluding infections) defined as number of AEs categorized by Medical Dictionary for Regulatory Activities (MedDRA) preferred terms, seriousness, and severity, divided by the number of infusions.
- Number/proportion of participants who develop positive titer (≥ 1:160) of binding or neutralizing antibodies to recombinant human hyaluronidase (rHuPH20).

Mode of product administration

- Infusions (Epoch 2)
  - Number of infusions per month.
  - Number of infusion sites (needle sticks) per infusion/month.
  - Duration of infusion.
  - Maximum infusion rate/site.
  - Infusion volume/site.
  - Number/proportion of infusions discontinued, slowed, or interrupted owing to an AE.
- Number of weeks to reach final dose interval (3 weeks or 4 weeks) (Epoch 1).
- Proportion of participants who achieve a treatment interval of 3 or 4 weeks in Epoch 2.
- Proportion of participants who maintain a treatment interval of 3 or 4 weeks in Epoch 2 for 12 months.

Health-related quality of life (HRQoL)

- Assessment of HRQoL questionnaires
    - Pediatric Quality of Life Inventory (Varni et al., 1999).
    - EuroQol five dimensions questionnaire (Rabin and de Charro, 2001).

Treatment preference and satisfaction

- Assessment of Life Quality Index (Daly et al., 1991; Nicolay et al., 2005).
- Assessment of Treatment Satisfaction Questionnaire for Medication (Bharmal et al, 2009).
- Assessment of Treatment Preference Questionnaire (internally developed by the study sponsor).

Healthcare Resource Utilization

- Days not able to go to school or work, or to perform normal daily activities owing to infection or other illnesses per participant-year.
- Days on antibiotics per participant-year.
- Number of hospitalizations, indication for the hospitalization (infection or non-infection), and days hospitalized per participant-year.
- Number of acute physician visits (office and emergency room) owing to infection or other illnesses per participant-year.

Definitions

Acute serious bacterial infections (ASBIs)

- ASBIs were defined based on US Food and Drug Administration guidance for industry (US Department of Health and Human Services, 2018) and the European Medicines Agency guideline on the clinical investigation of human normal immunoglobulin for subcutaneous and/or intramuscular administration (Committee for Medicinal Products for Human Use, 2015).
- The following bacterial infections were considered ASBIs and were diagnosed based on symptoms, physical findings, and laboratory test results (and imaging studies where applicable):
    - Bacteremia/sepsis.
    - Bacterial meningitis.
    - Osteomyelitis/septic arthritis.

    - Bacterial pneumonia.
    - Visceral abscess.

Adverse event seriousness

- A serious adverse event was defined as an untoward medical occurrence that at any dose met one or more of the following criteria:
    - Outcome was fatal/resulted in death.
    - Life-threatening – defined as an event in which the subject was, in the judgment of the investigator, at risk of death at the time of the event.
    - Required inpatient hospitalization (any inpatient admission, regardless of length of stay) or resulted in prolongation of an existing hospitalization.
    - Resulted in persistent or significant disability/incapacity (i.e. a substantial disruption of a person's ability to conduct normal life functions).
    - Congenital anomaly/birth defect.
    - Medically important event, i.e. a medical event that may not be immediately life-threatening or result in death or require hospitalization, but which may jeopardize the patient or may require medical or surgical intervention to prevent one of the other outcomes listed above.

Adverse event severity

The investigator assessed the severity of each AE using their clinical expertise and judgment based on the most appropriate description below:

Mild

- The AE was a transient discomfort and did not interfere in a significant manner with the patient's normal functioning level.
- The AE resolved spontaneously or may have required minimal therapeutic intervention.

Moderate

- The AE produced limited impairment of function and may have required therapeutic intervention.
- The AE produced no sequela/sequelae.

Severe

- The AE resulted in a marked impairment of function and may have led to temporary inability to resume usual life pattern.
- The AE produced sequela/sequelae, which required (prolonged) therapeutic intervention.

Adverse event causality

Causality is a determination of whether there is a reasonable possibility that the study drug is etiologically related to/associated with the AE. For each AE, the investigator assessed the causal relationship between the study drug and the AE using their clinical expertise and judgment according to the most appropriate algorithm for the circumstances of the AE (see below). These causality definitions were also used to assess the relationship of an AE with a study-related procedure, if necessary.

Not related (both circumstances must be met)

- AE due to underlying or concurrent illness, complications, concurrent treatments, or effects of concurrent drugs.
- AE not associated with the study drug (i.e. does not follow a reasonable temporal relationship to the administration of the study drug or has a much more likely alternative etiology).

Unlikely related (either one or both circumstances are met)

- Has little or no temporal relationship to the study drug.
- A more likely alternative etiology exists.

Possibly related (both circumstances must be met)

- Follows a reasonable temporal relationship to the administration of the study drug.
- An alternative etiology is equally or less likely compared to the potential relationship to the study drug.

Probably related (both circumstances must be met)

- Follows a strong temporal relationship to the administration of the study, which may include but is not limited to the following:
    - Reappearance of a similar reaction upon re-administration (positive re-challenge).
    - Positive results in a drug sensitivity test (skin test, etc.).

    - Toxic level of the study drug as evidenced by measurement of the study drug concentrations in the blood or other bodily fluid.
- Another etiology is unlikely or significantly less likely.
- For events assessed as ‘not related’ or ‘unlikely related’ and occurring within 72 hours after completion of study drug administration, the investigator shall provide the alternative etiology.

References

Bharmal M, Payne K, Atkinson MJ, Desrosiers M-P, Morisky DE, Gemmen E. Validation of an abbreviated Treatment Satisfaction Questionnaire for Medication (TSQM-9) among patients on antihypertensive medications. Health Qual Life Outcomes. 2009;7:36.

Committee for Medicinal Products for Human Use. Guideline on the clinical investigation of human normal immunoglobulin for subcutaneous and/or intramuscular administration (SCIg/IMIg).EMA/CHMP/BPWP/410415/2011 rev 1. 2015. [Internet]. https://www.ema.europa.eu/en/documents/scientific-guideline/guideline-clinical-investigation-human-normal-immunoglobulin-subcutaneous-andor-intramuscular-administration-scigimig-revision-1_en.pdf. (Accessed June 13, 2024).

Rabin R, de Charro F. EQ-5D: A measure of health status from the EuroQol Group. Ann Med. 2001;33:337–43.

US Department of Health and Human Services, Food and Drug Administration and Center For Biologics Evaluation and Research. Guidance for industry: safety, efficacy, and pharmacokinetic studies to support marketing of immune globulin intravenous (human) as replacement therapy for primary humoral immunodeficiency. 2008. [Internet]. https://www.fda.gov/media/124333/download. (Accessed June 13, 2024).

Varni JW, Seid M, Rode CA. The PedsQL: measurement model for the pediatric quality of life inventory. Med Care. 1999;37:126–39.
