# Supplementary material for: Efficacy, Safety, Tolerability, and Serum IgG Trough Levels of Hyaluronidase-Facilitated Subcutaneous Immunoglobulin 10% in US Pediatric Patients with Primary Immunodeficiency Diseases
Source: J Clin Immunol. 2025 Mar 14;45(1):81. doi: 10.1007/s10875-025-01862-6 (PMC11909037; doi:10.1007/s10875-025-01862-6)
Supplement: Supplementary file 3 — Supplementary Material 3 [file 10875_2025_1862_MOESM3_ESM.pdf]

Efficacy, Safety, Tolerability, and Serum IgG Trough Levels of Hyaluronidase-facilitated Subcutaneous Immunoglobulin 10% in US Pediatric Patients with Primary Immunodeficiency Diseases

Journal of Clinical Immunology

Niraj C. Patel • Jolan E. Walter • Richard L. Wasserman • Arye Rubinstein • Suthida Kankirawatana • Meagan W. Shepherd • Erin Greco • Zhaoyang Li • Sharon Russo-Schwarzbaum • Shumyla Saeed-Khawaja • Barbara McCoy • Leman Yel

Corresponding author:
Leman Yel, M.D.
lyel@uci.edu

Supplementary Results

Table S1 Rate of all infections

	**All participants (*N* = 44)**
Total number of infections	161
Number (%) of participants with infections	34 (77.3)
Rate of infections per participant-year (upper CI)	3.12 (3.95)
Number (%) of participants with infection AEs[a]	
Sinusitis	18 (40.9)
Upper respiratory tract infection	9 (20.5)
Viral upper respiratory tract infection	9 (20.5)
Streptococcal pharyngitis	7 (15.9)
Influenza	6 (13.6)
Otitis media	6 (13.6)
Acute sinusitis	5 (11.4)
Ear infection	4 (9.1)
Gastroenteritis (viral)	4 (9.1)
Bronchitis	3 (6.8)
Conjunctivitis	3 (6.8)
Gastroenteritis	3 (6.8)
Molluscum contagiosum	3 (6.8)
Otitis externa	3 (6.8)

Pharyngitis	3 (6.8)
Chronic sinusitis	2 (4.5)
Hordeolum	2 (4.5)
Impetigo	2 (4.5)
Nasopharyngitis	2 (4.5)
Respiratory tract infection (viral)	2 (4.5)
Urinary tract infection	2 (4.5)
Abscess (limb)	1 (2.3)
Adenovirus infection	1 (2.3)
Bronchitis (viral)	1 (2.3)
Bullous impetigo	1 (2.3)
Clostridium difficile colitis	1 (2.3)
Conjunctivitis (bacterial)	1 (2.3)
Conjunctivitis (viral)	1 (2.3)
COVID-19	1 (2.3)
Cystitis	1 (2.3)
Fungal skin infection	1 (2.3)
Gastroenteritis (sapovirus)	1 (2.3)
Gastrointestinal (viral infection)	1 (2.3)
Genital candidiasis	1 (2.3)
Herpes simplex	1 (2.3)
Lymphadenitis (viral)	1 (2.3)
Oral herpes	1 (2.3)
Otitis media (acute)	1 (2.3)
Otitis media (chronic)	1 (2.3)
Periorbital cellulitis	1 (2.3)

[a]Infections are AEs (preferred terms) within the system organ class equal to infections and infestations

AE, adverse event; *CI*, confidence interval
